# Teaching removable partial denture design: ‘METACIEL’, a novel digital procedure

**DOI:** 10.5116/ijme.5a5b.2b0d

**Published:** 2018-01-26

**Authors:** Guillaume Bonnet, Cindy Lance, Marion Bessadet, Faleh Tamini, Jean-luc Veyrune, Olivier Francois, Emmanuel Nicolas

**Affiliations:** 1 Université Clermont Auvergne, CROC, F-63000 Clermont-Ferrand, France and CHU Clermont-Ferrand, Service d’Odontologie, F-63003 Clermont-Ferrand, France; 2 Division of Prosthodontics and Restorative Dentistry, Faculty of Dentistry, McGill University, Montreal, QC, Canada

**Keywords:** Teaching, removable partial denture, METACIEL, digital procedure

## To the Editor

Nowadays, academic teaching relies more and more on digital principles, such as e-learning. In dentistry, digital programs could facilitate the learning of features hard to understand. In the Fixed Dental Prosthesis domain, learning tooth preparation is challenging as well as evaluating students’ progress. In this context, companies, specialized in CAD/CAM technology (Computer Aided Design/Computer Aided Manufacturing), developed specific teaching software to evaluate the quality of dental preparation realized on physical simulation model, with a high number of manageable parameters. Assessment of such system proved to be of great educational value,^1^^,^^2^ and this software is also used for teaching oral cavities in restorative dentistry and dental anatomy.^3^^,^^4^ It turned out to be a very interesting preclinical teaching tool.^5^^,^^6^ In the Removable Partial Denture domain (RPD), learning framework design is challenging and lack of knowledge in this area could lead to many clinical consequences.^7^ No dedicated digital teaching procedure is yet available. RPD design software packages are available and are being used by commercial prosthetic laboratories, especially related to 3D printing of ‘resin patterns’ or the metal framework itself. However, this software was not developed for educational purpose, and it became obvious that a web application for teaching framework design needed to be developed. Therefore, the Clermont Auvergne University has been conceiving in recent years a specific educational application, METACIEL, from which the first available version is used within the prosthesis teaching framework.

The development of METACIEL followed the steps outlined by Johnson and Schleyer^8^ in 2003 for the development of quality educational software: (1) ANALYSIS: during this step, the project objectives, information sources, and requirements were defined. METACIEL has been developed in view of a dual purpose: teaching (students are able to evaluate themselves at any of the RPD design process stage) and evaluation (scores obtained by the students are accessible to teachers); (2) DESIGN: this step included the production of diagrams illustrating the interface, their sequence of appearance and configuration in space and the redaction of the starting guide for proper utilisation of the application; (3) DEVELOPMENT: two actions were carried out during this step. Firstly, images of the main types of edentulous cases (Kennedy classification) using scans of resin self-made models.^9^ Digital pictures of each component of RPD framework designs were also produced. This step was completed by adding multiple choices questions related to RPD basic knowledge and their corresponding answers. Secondly, computer programming allowed implementation of METACIEL on an opensource web server. The main virtual partially edentulous cases were developed in 3D.^9^ The connection to a specific network has enabled the establishment of a web server for the site's development and use. An online tutorial is also available on the METACIEL CROCUCA web site. Each case is a 3D representation of the oral cavity, with the possibility to move the model in space. It is also possible to visualize the occlusion interface, the framework design progress and the denture insertion axis (with visualization of undercut areas). All virtual METACIEL cases are divided into three parts: (1) a drawing area: the student must select each item in the menu and place them on the image through the “click and drag” action button; (2) a section for quiz and answers; and (3) the correction image of the RPD design step that allow the student to self-evaluate.

As of today, the main phase of METACIEL development is completed. However, further improvements should be conceptualized, including, for example, additional types of partially edentulous cases and random onsets of quizzes for the students. Moreover, METACIEL was designed according to a specific process which could be modulated, on request, for other precept. Besides METACIEL usage on a computer, digital tablet or smartphone applications are currently under development. Several Francophone universities (University of Nice Sofia Antipolis (France), University of Paul Sabatier (Toulouse III France), University of Dakar Cheikh Anta Diop (Senegal)) are in the process of integrating METACIEL in their educational curriculum. Simultaneously, the English version of the web application, “Metasky,” will be developed in collaboration with an Anglophone university (McGill University). Further testing and systematic use by students within the French universities and/or faculties will optimize the current version. By including the evaluation from a wide number of dental students from different countries, assessment of the educational outcome of this digital tool will be a more thorough. METACIEL fits perfectly within the two development axes in modern oral care. Firstly, it takes part in the new digital education system used in dentistry, such as use of simulators.^10^ To this day, these developments mainly focused on studying dental bur usage, but applications in the removable prosthesis domain are possible.^11^ Secondly, the use of CAD/CAM technology in dentistry has grown significantly over the past decade, but with yet a relatively recent use in the removable prosthesis domain. It was only recently that the first CAD/CAM system designed for removable prosthesis was put into use and enabled a virtual framework conception by computer design using specialized software. The virtual data are then transferred to a tool machine that enables production of the frame. METACIEL application is not designed to produce metal frame and cannot be used as a CAD/CAM technology, but it follows, in a very simplified way, the modelling steps from the CAD/CAM software. METACIEL is thus innovative educational web application as it integrates the learning of CAD/CAM technology within the dentistry curriculum. At the end of its cursus, a student should be able to handle the complete digital system: taking a digital impression, designing the virtual framework and shipping online to the dental technician.

In conclusion, METACIEL could to be a good educational tool to RPD design teaching. Indeed, implementing Information and Communication Technologies support for education (eICT) within the dental cursus could enable future dentists to grasp conceptual, difficult to apprehend, procedures especially in the dental prosthesis domain. Furthermore, it could be used to introduce 3D RPD modelling knowledge, which, via CAD/CAM procedure usage, is going through a period of significant growth.

### Acknowledgements

This project was supported by the UNF3S – UNSOF numerical university in order to enhance and diffuse the software amongst the francophone dental community.

### Conflict of Interest

The authors declare that they have no conflict of interest.
